# Persistent positive anomalies in geopotential heights drive enhanced wildfire activity across Europe

**DOI:** 10.1098/rstb.2023.0455

**Published:** 2025-04-17

**Authors:** Kerryn Little, Dante Castellanos-Acuna, Piyush Jain, Laura Graham, Nicholas Kettridge, Mike Flannigan

**Affiliations:** ^1^ School of Geography, Earth and Environmental Sciences, University of Birmingham, Birmingham B15 2TT, UK; ^2^ Department of Renewable Resources, University of Alberta, Edmonton, Alberta T6G 2E3, Canada; ^3^ Northern Forestry Centre, Canadian Forest Service, Natural Resources Canada, Edmonton, Alberta T6H 3S5, Canada; ^4^ Biodiversity, Ecology and Conservation Group, International Institute for Applied Systems Analysis, Vienna 2361, Austria; ^5^ Department of Natural Resource Science, Thompson Rivers University, Kamloops, British Columbia V2C 0C8, Canada

**Keywords:** wildfire, heatwave, synoptic, blocking, fire weather, Europe

## Abstract

Persistent positive anomalies (PPAs) in 500 hPa geopotential height are upper-air circulation patterns associated with surface heatwaves, drought and fuel aridity. We examined the association between PPA events and surface fire weather and burned area at a pan-European level. Europe-wide, extreme fire weather and wildfires were on average 3.5 and 2.3 times more likely to occur concurrently with a PPA, respectively. PPAs were associated with 45% of pan-European area burned between March and October 2001–2021, and there was a latitudinal increase of up to 63% in the percentage of area burned during or up to 7 days following PPAs over Northern Europe. The burned area was highest up to one week following PPA presence, and fuel moisture indices from the Canadian Fire Weather Index System lagged behind peak PPA strength, demonstrating the role of PPAs in pre-drying fuels. Our findings highlight opportunities for developing early warning systems of wildfire danger, having implications for wildfire awareness and preparedness, informing policy and wildfire management decisions like early mobilization and resource sharing initiatives across Europe.

This article is part of the theme issue ‘Novel fire regimes under climate changes and human influences: impacts, ecosystem responses and feedbacks’.

## 1. Introduction

Recent extreme wildfire seasons across Europe have demonstrated how long-duration synoptic weather patterns promote synchronous surface fire weather extremes that can strain firefighting resources and even overwhelm response capabilities [1]. Global climate change is increasing the risk of co-occurring fire weather extremes across large geographic regions [2].

In the Northern Hemisphere mid-latitudes, surface weather is driven by the west-to-east progression of synoptic-scale weather systems. Synoptic scale refers to atmospheric circulation conditions operating over time periods of approximately 2−10 days and spatial scales from hundreds to thousands of kilometres [3]. Anticyclonic blocking highs and ridges are areas of high pressure that are near stationary and disrupt the usual zonal airflow for an extended period, leading to hot, dry surface weather [4]. Where these systems persist, they can lead to extreme events such as heatwaves and drought [5–7]. Persistent positive anomalies (PPAs) in 500 hPa geopotential heights are associated with a range of blocking patterns, characterized by weakened or reversed zonal flow and persistent, large positive anticyclonic anomalies [8,9]. Warming and drying of descending air parcels during PPAs result in extreme surface temperatures, which may be amplified by land–atmosphere feedbacks including soil moisture deficits and snow cover changes [5–7]. During the northern hemisphere summer, PPAs have been associated with notable heatwaves and droughts throughout Europe, including the 2003 heatwave that led to an estimated 70 000 deaths in Europe [10].

PPAs are also important for wildfires. Associated extremes in surface fire weather are conducive to the pre-drying of fuels, ignition of wildfires and their subsequent behaviour [11–13]. Moreover, atmospheric subsidence (descending warm, dry air) can lead to poor surface air quality from wildfire smoke trapped at the surface, with the potential to impact densely populated regions such as the air pollution events in Russia during the 2010 heatwave [14]. PPAs have recently been directly associated with wildfire activity in western North America [11]. Wildfires were on average seven times more likely to start during PPAs and were even more likely at higher latitudes (eightfold increase above 50°N). To our knowledge, whether these relationships also hold for the diversity of fire behaviour and human-dominated ignitions across Europe has not yet been examined.

Weather can influence fire occurrence and behaviour at different spatiotemporal scales. Relationships between surface fire weather and wildfire activity have been well documented at global [15] and regional levels [16]. Operationally, surface weather observations are used to monitor wildfire danger; however, their high spatiotemporal variability makes it difficult for numerical weather prediction systems to generate reliable forecasts beyond the short term (4–7 days). Longer-varying upper-air synoptic-scale circulation patterns, by contrast, can be more reliably predicted in the medium range (+10 days) as they do not include the complexity of land surface and boundary layer effects. Assessing synoptic indicators of wildfire activity in addition to surface fire weather may prove more insightful for forecasting fire danger. Previous research found that the error of a 10 day synoptic forecast was equivalent to a one-day convective-scale forecast [17]. Understanding how PPAs impact wildfire across Europe may therefore open opportunities to improve our near-to-medium range forecasting of dangerous wildfire conditions. Improved forecast lead times aid effective decision-making, including wildfire preparedness and resource allocation/movement. Advanced warnings of elevated wildfire risk are useful both within and across agencies and countries.

Previous research has examined linkages between synoptic circulation patterns and wildfire activity in Europe, including in-depth studies on specific wildfire events and seasons [18,19] and synoptic weather typing (classification of large-scale atmospheric patterns) for wildfires in specific regions. Of the latter, most research has focused on Southern Europe, including Catalonia, Spain [20,21], France [22], Greece [23] and Portugal [24]. Very few studies have examined synoptic weather patterns and their relationship with fire for other regions of Europe (e.g. European boreal zone [25] and the Central European Alps [26]) and even less research has considered these relationships at a pan-European scale. [27] recently related extreme fire weather to blocking over northern Europe and subtropical ridging over southern Europe. Their findings highlighted the importance of positive upper air geopotential height anomalies (Z500) at a continental scale. Classifying large-scale fire weather extremes and the relationship with fire activity using an event-based paradigm, such as PPAs, has not yet been explored for Europe. There is scope for a pan-European analysis of the role of PPAs in driving wildfires, as the large spatial and temporal nature of PPAs may constrain wildfire response capabilities across political borders for an extended period. Such a scenario occurred during the exceptional 2023 wildfire season in Canada that experienced persistent blocking ridges in both the west and east, stretching firefighting resources beyond their limits across the country [28]. Understanding the spatiotemporal extent of PPAs across Europe can provide insights for coordinating pan-European wildfire response and resource allocation.

There is also significant uncertainty surrounding how large-scale synoptic weather patterns will be affected by future changes in climate. There is some evidence that the conditions conducive to PPA formation will become more common in the future with weakening of the jet stream [29]. High-latitude anthropogenic warming during summer may lead to increased occurrence of double jets between atmospheric blocks [6]. However, whether we are currently observing a weakened jet stream is still actively debated [30]. Other models predict a decrease in blocking frequency [31]. Representation of atmospheric dynamics such as PPAs in climate models is poor, and the physical mechanisms of PPA response to anthropogenic warming are still debated [32,33]. As such, it is critical to first understand how atmospheric circulation patterns like PPAs drive wildfire activity in the present.

We examine the association between PPAs and wildfire across Europe between March and October 2001–2021 by addressing the following research questions: (i) what are the characteristics of European PPA events? And (ii) to what extent are PPAs associated with surface fire weather and wildfire activity across Europe?

## 2. Methods

### (a) Study region

**Figure 1.**
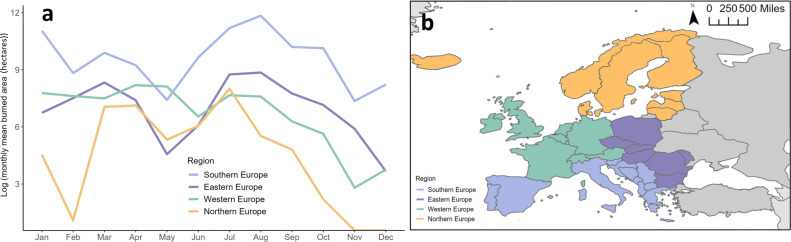
(a) Average monthly log-transformed burned area by region.(b) Map of Europe and the four geographical regions used to associate PPAs with surface fire weather and burned area. Countries with no 2001−2021 MODIS wildfire records were excluded from the regional analyses of PPA–burned area associations.

We applied a bounding box of 30°N to 75°N and −50°E to 60°E to detect and track PPA events at a pan-European level [34]. Estimated *ca* 96.5 ± 0.9% of burned area across Europe is started by anthropogenic activities, while lightning ignitions can be regionally important, such as for the high latitudes of Scandinavia. Fire regimes (area burned and seasonality) within Europe vary across four main regions in Western Europe (figure 1a; electronic supplementary material, figure S1), but climate and land use change are impacting wildfire occurrence and behaviour across the continent [35,36]. Southern European countries are traditionally fire-prone but are experiencing new fire phenomena and extremes associated with climate, land use and social change [35]. The temperate and boreal regions of Western and Northern Europe are emerging fire-prone regions that contain globally critical carbon stores and are experiencing increasing wildfire risk [37,38].

We present PPA–fire associations by four geographical regions: Northern, Southern, Eastern and Western Europe (figure 1b). In doing so, we aim to capture the synoptic signal of PPA–fire relationships, despite the variability of wildfire occurrence across Europe associated with local effects like vegetation cover, fire management strategies and ignition sources. We excluded three far Eastern European countries (Republic of Türkiye, Ukraine and Belarus) from our analyses owing to the large number of agricultural fires that could not be accounted for within the scope of this research [39,40].

### (b) Data

#### (i) Atmospheric data

We used gridded 500 hPa geopotential height (Z500) and surface variables from the European Centre for Medium-Range Weather Forecasts ERA5 global reanalysis dataset, extracted for the study area between March and October 2001−2021 [41]. Hourly surface variables included were accumulated precipitation, 2 m air temperature and relative humidity and 10 m wind speed. From these data, we calculated hourly vapour pressure deficit (VPD), which is an absolute measure of the ability of the atmosphere to extract moisture and hence is related to vegetation water stress [42]. Noon (local grid-cell time zone) values of the ERA5 surface variables were extracted to calculate the Canadian Fire Weather Indices [43]. The Canadian Fire Weather Index System (CFWIS) describes the effects of surface weather on fuel moisture and fire behaviour; the outputs comprise the fine fuel moisture code (FFMC), duff moisture code (DMC), drought code (DC), initial spread index (ISI), build-up index (BUI) and the fire weather index (FWI). The first three codes refer to the moisture conditions of the litter, top organic layer and deeper soil layer with increasing equilibrium nominal times of 16 h, 15 days and 52 days, respectively [44]. The last three codes are fire behaviour indices, describing the expected rate of spread (ISI), amount of fuel available (BUI) and an overall rating of fire intensity (FWI). We also calculated the hot–dry–windy index (HDWI), which is the product of the maximum VPD and wind speed that occurs in the lowest 50 hPa layer of the atmosphere [45]. The HDWI captures dry air masses and winds that might mix down to the surface to drive fire growth. We defined extreme fire weather (FWIx) as days exceeding the 95th percentile of FWI values for each grid cell for each month between March and October. ERA5 atmospheric variables were aggregated from 0.25 × 0.25 to 1 × 1 degree spatial and daily average temporal resolutions because synoptic patterns, including PPAs, are sufficiently resolved at this resolution [11,46,47]. Anomalies of surface variables were calculated by subtracting daily values from the long-term climatological mean for the study period (2001−2021).

#### (ii) Wildfire data

We obtained burned area records for our study region from the database provided by the European Forest Fire Information System (EFFIS) for the period March–October available from 2001 to 2021 [48]. In selecting this time window, we capture the active fire seasons of most biomes in Europe [49,50], although we recognize this may exclude winter wildfires that can occur outside of this primary window of interest [51]. The EFFIS burned area product produces polygons from the processing of Moderate Resolution Imaging Spectroradiometer (MODIS) imagery from the Aqua and Terra satellites at 250 m spatial resolution and it has been further refined by Sentinel-2 imagery since 2018. Prior to 2006, burned area was mapped using satellite imagery from the Wide Field Sensor instrument on the Indian Remote Sensing satellites and initial dates were defined *a posteriori* [52,53]. We omitted any wildfires where initial date could not be determined prior to 2006. As we do not analyse interannual trends in PPA–fire relationships or individual wildfire events, any methodological changes in the burned area product are unlikely to impact our results. The EFFIS burned area database captures wildfires larger than 30 ha across Europe, representing *ca* 95% of the total annual burned area in the European Union, which makes it suitable for analysing large wildfires at the pan-European level [54]. We summed daily burned area polygons within each 1 × 1 degree grid cell, where the date used is the initial date of the wildfire detection and burned area is total burned area (ha) per grid cell. We did not apply a burned area threshold to our main analyses (aside from the size detectable by MODIS) owing to the lower total number of available records and smaller overall wildfire size in some regions. We acknowledge that this may result in some biases at the lowest burned areas depending on detection efficiency and the presence of false negatives.

### (c) Persistent positive anomalies identification

We used the PPA algorithm of [11] (adapted from [55]) to detect and track PPA events, adjusting the minimum size from 80 000 to 40 000 km^2^ to fit the European context. A brief description of the algorithm is as follows (for full details see [11]). We first calculated daily Z500 anomalies for each grid cell and applied a 5 day moving mean and latitude correction factor to properly account for atmospheric energy dispersion [9]. Because we are most interested in PPA events during the main wildfire seasons when pressure gradients are weaker compared with winter, we used the daily varying mean standard deviation of the geopotential height anomaly in a four week moving window to define a seasonally varying threshold for magnitude. Grid cells that exceeded a given threshold for both magnitude and duration criteria were identified as PPA grid cells. Here, we used a minimum magnitude of 1 × s.d. and minimum duration threshold of 5 days. Subsequently, the geometric centroid of spatially contiguous PPA grid cells was tracked until they reached a size of 40 000 km^2^, at which point they were labelled as an individual PPA event. We also assessed the sensitivity of the algorithm to different thresholds. While the algorithm was relatively insensitive to magnitude and size, it was affected most by the duration, so we selected 5 days to exclude shorter events.

### (d) Odds ratio analysis

We categorized each day as either PPA–fire, PPA–nofire, noPPA–fire or noPPA–nofire for each grid cell in our spatial domain (table 1) as follows. We defined PPA–fire days as the presence of a PPA during or up to 7 days prior to a wildfire event (initial day of burned area recorded) for each grid cell, with the lag accounting for the role of PPA conditions in pre-drying fuels that may subsequently ignite. We assessed the sensitivity of our analyses to different sensible time lags, finding no major differences in our results (electronic supplementary material, table S1). We used the same approach to define PPA–extreme fire weather days as PPA–FWIx, PPA–noFWIx, noPPA–FWIx or noPPA–noFWIx for each grid cell; however, we confined PPA–FWIx days to the presence of a PPA on the same day as extreme FWI recorded (i.e. zero time lag).

**Table 1.** Each daily grid cell record is categorized as a PPA–fire, PPA–nofire, noPPA–fire or noPPA–nofire day to define PPA–fire associations and a PPA–FWIx, PPA–noFWIx, noPPA–FWIx or noPPA–noFWIx day to define PPA–FWIx associations.

category	definition
PPA–fire	presence of a PPA during or up to 7 days prior to a wildfire event
PPA–nofire	presence of a PPA in a grid cell is not coincident with a wildfire event during or up to 7 days following the PPA
noPPA–fire	a wildfire event is recorded but is not associated with the presence of a PPA
noPPA–nofire	no PPA or wildfire activity recorded
PPA–FWIx	presence of a PPA coincident with extreme surface FWI values (above the 95th percentile)
PPA–noFWx	the presence of a PPA in a grid cell is not coincident with extreme FWI values
noPPA–FWIx	extreme FWI is recorded but not associated with the presence of a PPA
noPPA–noFWx	no PPA or extreme FWI recorded

The odds ratio (*OR*) is a measure of the likelihood that an outcome (extreme fire weather or wildfire activity) will occur given the presence of an exposure (PPA), compared with the likelihood of the outcome occurring without an exposure [56]. We calculated the *OR* to determine the association between PPAs and wildfire activity, where *a* = PPA – fire, *b* = PPA – nofire, *c* = noPPA – fire and *d* = noPPA – nofire (equation (2.1)). Using the same equation, we calculated the *OR* for the association between PPAs and extreme fire weather, where *a* = PPA – FWIx, *b* = PPA – noFWIx, *c* = noPPA – FWIx and *d* = noPPA – noFWIx.


(2.1) $$OR=\frac{a\;\times d}{b\;\times \;c}$$

We used a partial Haldane correction to address the issue of cells containing zero values leading to errors in the reported *OR* and to minimize the estimation bias of the *OR*. This involves adding a correction factor to all values in the contingency table where there are any zero values [57]. A correction factor of +2 can be used to minimize estimation bias of small sample sizes such as in this dataset [56]. We applied a correction factor of +2 to all the values in the *OR* calculation where grid cells contained a zero value for (*c*) (i.e. all fire activity occurred during a PPA) to obtain a sensical value of the PPA–fire association. We similarly applied a correction factor of 2 where normal *OR* values were obtained (i.e. cells with no zero values) for consistency. Grid cells with no observed fire within the study area were excluded from the analysis of PPA–fire. We calculated the *OR* for each grid cell and month using counts of the daily timeseries of records as defined in table 1. We presented summary statistics of the grid-cell level *OR* by region (and by country in the electronic supplementary material) to reduce the sensitivity of the reported *OR* to individual grid cells. We also calculated the *OR* for the association between PPAs and extreme fire weather following the above approach.

### (e) Statistical analyses

We used linear regression models for each grid cell by month to examine differences in surface weather anomalies between PPA and non-PPA days. In these models, the surface weather anomaly was the dependent variable and the independent variable was a two-level categorical variable detailing whether a day is PPA or non-PPA. The residuals of the models were normally distributed, as assessed visually by *Q*–*Q* plots, and they did not display heteroscedasticity. We presented the monthly average *t*-statistic for the regression slope from the linear model for each grid cell, where positive values depict larger surface anomalies on PPA days. We used the *t*-statistic of the slope rather than the slope itself as this means the values are comparable.

We examined the lead–lag relationship between maximum PPA strength and daily surface anomalies to examine the response of surface conditions to PPAs. The PPA area on the day of maximum PPA strength was used for calculating spatially averaged surface anomalies for 15 days prior to and following the day of maximum strength. We calculated PPA strength as the daily summed area-weighted magnitude (i.e. the 500 hPa anomaly; gpm km^2^).

We generated descriptive statistics to summarize PPA events in Europe and their association with wildfire activity as per [11]. The PPA algorithm and all statistical analyses were carried out in R v. 4.1.2 [58], using packages igraph [59], Raster [60], Terra [61] and zyp [62].

## 3. Results

### (a) Characteristics of European persistent positive anomalies

**Figure 2.**
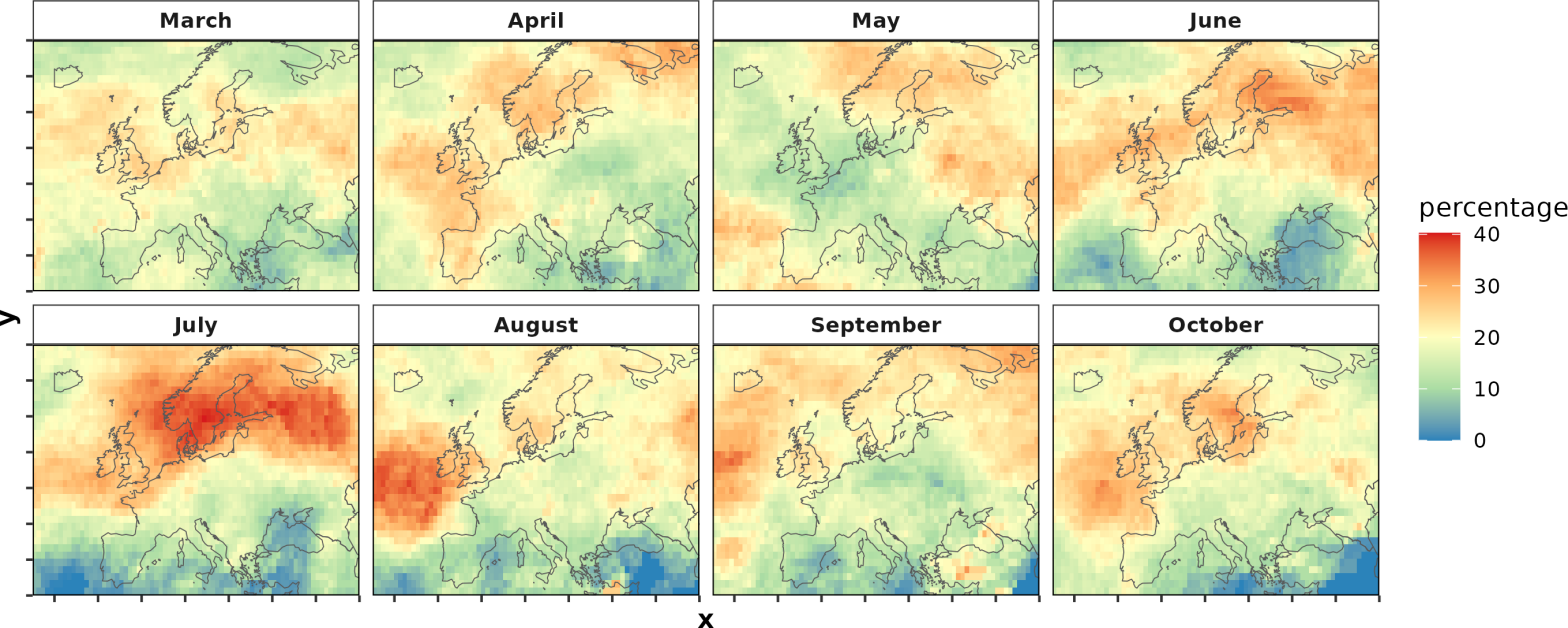
Average monthly percentage of PPA days for each 1 × 1 degree grid cell across the pan-European spatial domain of 30°N to 75°N and −50°E to 60°E, 2001−2021.

We identified 643 PPA events between March–October 2001−2021 across Europe (an average of 30.6 events per year) and the mean event duration was 12.1 days (electronic supplementary material, figure S2). July and August had the highest percentage of PPA days, which were centred over Scandinavia and the North Atlantic Ocean, respectively (figure 2).

### (b) Persistent positive anomalies and surface fire weather

**Figure 3.**
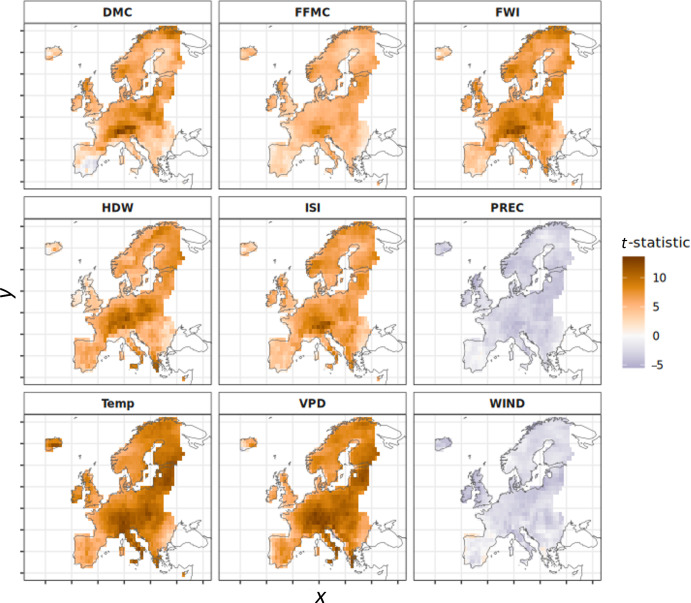
*T*-statistic averaged across the linear regression models for the summer months of June, July and August comparing surface anomalies between PPA and non-PPA days. Positive values (orange) denote grid cells where surface anomalies are higher on PPA days than on days where there was no PPA. Negative values (purple) denote grid cells where surface anomalies are lower on PPA days than non-PPA days.

Anomalies of surface air temperature (Temp) and VPD are greater for PPA days than non-PPA days, while wind speed anomalies (WS) and precipitation (PREC) are lower during PPAs. Anomalies are greatest in Central and Northern Europe, while the relationship is weaker in Southern Europe (figure 3). The FWI, FFMC, ISI and HDW fire weather indices are higher for PPA days. DMC anomalies are greater on PPA days for parts of central and Northern Europe but are lower for parts of Southern Europe compared with non-PPA days. Differences between PPA and non-PPA days are stronger during summer than spring months; see electronic supplementary material, figures S3 and S4 for a full monthly breakdown of all variables.

**Figure 4.**
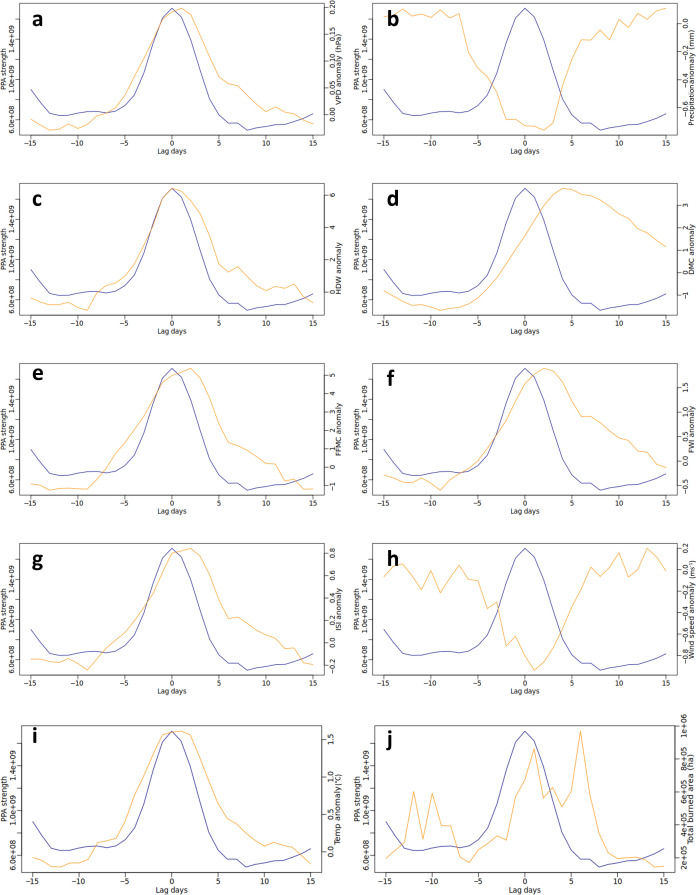
Lead–lag relationship between PPA strength (gpm km^2^) and surface anomalies. Blue line = PPA strength with maximum strength on day 0. Orange line = average (a) VPD anomaly (hPa), (b) precipitation anomaly (mm), (c) HDWI anomaly (hPa m s^−1^), (d) DMC anomaly, (e) FFMC anomaly, (f) FWI anomaly, (g) ISI anomaly, (h) wind speed anomaly (m s^−1^), (i) temperature anomaly (°C) and (j) total burned area (ha) for the maximum PPA strength area 15 days either side of maximum PPA strength.

Daily VPD and midday temperature anomalies increase with strengthening PPA, while accumulated precipitation decreases (figure 4). The maximum of surface anomalies occurs alongside maximum PPA strength, except for wind speed, which reaches a low one day following maximum PPA strength and precipitation, which reaches a low 2 days following maximum PPA strength. The CFWIS components have a shifted lag following peak PPA strength, which is consistent with the longer response times of the DMC and DC and continued drying within the soil following the peak of the event (electronic supplementary material, figure S5). Wind speeds are anomalously low during the maximum PPA strength and increase following the breakdown of the system, but there is a lag of several days for wind speeds to return to normal levels. The total burned area for each lag day shows spikes ahead of and following peak PPA strength, particularly on lag days 6−7, as well as a peak in burned area during the maximum PPA strength.

### (c) Persistent positive anomalies and extreme fire weather

**Figure 5.**
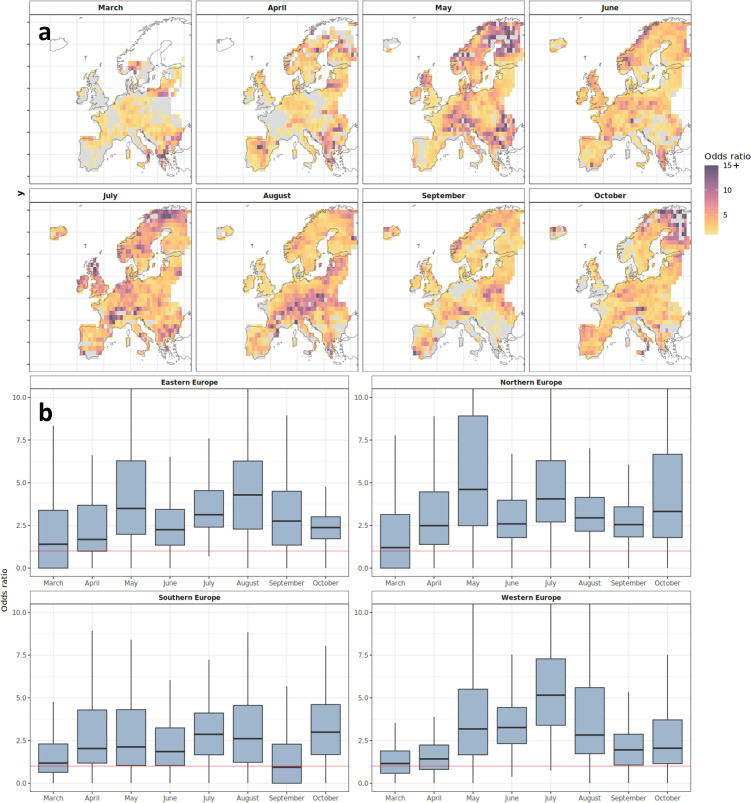
*OR* analysis showing the likelihood of a PPA occurring concurrently with extreme FWI (above the 95th percentile). *ORs* were calculated monthly for each grid cell (a) and summarized by region (b). *OR* > 1 (non-grey colour ramp in (a) and above red lines in (b)) indicate a higher likelihood of experiencing extreme FWI values concurrently with the presence of a PPA. Grey cells in (a) depict locations where extreme FWI values are less likely during PPAs, and white cells are locations with insufficient data to calculate an *OR*. The colour ramp in (a) is set to a maximum of 15, where *OR* > 15 are set to *OR* = 15 to allow for ease of interpretation.

The odds of the 95th percentile of FWI values being exceeded is an average of 3.5 times greater under PPA conditions. Extreme FWI values are most likely to coincide with PPA events for Northern (mean *OR* = 4.2), followed by Eastern Europe (mean *OR* = 3.4), Western (mean *OR* = 3.3) and Southern Europe (mean *OR* = 2.9). There is substantial monthly variability in the influence of PPAs on extreme fire weather (figure 5a,b). A more detailed breakdown of *ORs* by country and region can be found in electronic supplementary material, figure S6 and table S2.

### (d) Persistent positive anomalies and wildfire burned area

**Figure 6.**
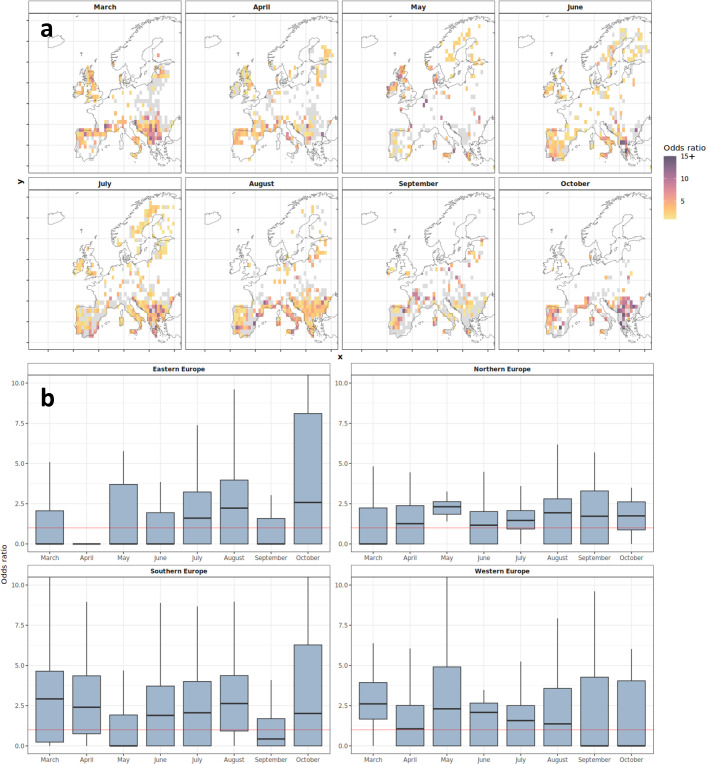
*OR* analysis showing the likelihood of burned area being recorded during or following (up to 7 days) PPA overlap. *ORs* were calculated monthly for each grid cell (a) and summarized by region (b). *OR* > 1 (non-grey colour ramp in (a) and above red line in (b)) indicates a higher likelihood of wildfires occurring during or following (up to 7 days) the presence of a PPA. Grey cells in (a) depict locations where wildfires are less likely during PPAs, and white cells are locations with insufficient data to calculate an *OR*. The colour ramp in (a) is set to a maximum of 15, where *OR* > 15 are set to *OR* = 15 to allow for ease of interpretation.

On average, the odds of a wildfire occurring concurrently with a PPA increase by a factor of 2.3 across the four regions of Europe between March and October. Our findings are spatiotemporally variable, reflecting the differences in wildfire regimes across Europe (figure 6a). We therefore also look at monthly and regional associations between PPAs and wildfire (figure 6b). Regionally, wildfires are most likely to occur concurrently with PPAs for Southern Europe (mean *OR* = 2.7), followed by Western (mean *OR* = 2.2), Eastern (mean *OR* = 2.0) and Northern Europe (mean *OR* = 1.5). In contrast, the percentage of burned area associated with PPAs was highest for Northern (63%) and Western Europe (53%), followed by Eastern (47%), then Southern Europe (44%). A more detailed breakdown of *ORs* and burned area associated with PPAs by region and country in addition to month is given in electronic supplementary material, figures S7–S9 and table S3. The monthly burned area associated with PPAs is highest in July for Northern Europe compared with October for Southern Europe (electronic supplementary material, figure S7). Overall, burned area is highest in the one week period following the presence of a PPA (electronic supplementary material, figure S10).

## 4. Discussion

PPAs are significant drivers of extreme fire weather and wildfire activity across Europe and their importance relative to other processes increases latitudinally from Southern to Northern Europe. Europe-wide, all regions were more likely to experience extreme fire weather and enhanced wildfire activity for all months between March and October for the years between 2001 and 2021, inclusive (except Eastern Europe wildfires in April).

### (a) Pan-European persistent positive anomalies climatology

We have characterized a climatology of spring and summer PPAs across Europe. Different methods for quantifying atmospheric blocking make direct between-study comparisons difficult. However, the patterns of PPA persistence across Europe that we detected agree with previous research, including peak blocking frequency across Scandinavia [47,55,63]. We detected a higher frequency of PPAs across Europe than those found by [11] for western North America (although our minimum PPA size was lower, which is likely a contributing factor). Higher blocking frequency in Europe compared with elsewhere in the Northern Hemisphere by a factor of 3−4 has also been found in other research [6,63].

### (b) Persistent positive anomalies and surface fire weather

Our pan-European assessment of the influence of PPAs on surface fire weather found that in general, surface fire weather is more extreme across Europe when a PPA is present, particularly for Northern and Eastern Europe. PPAs appear to be less important in driving extreme FWI values for Southern Europe. When FWI values are already high, such as during summer in Southern Europe, the FWI is most sensitive to wind speeds, which are not generally anomalous under a PPA. Thus, while the FWI may be high under PPAs, it may not become extreme [64]. This suggests that PPAs are more important in Northern Europe, while other processes—such as prolonged drought and strong wind events—may be more important in driving extreme surface conditions in Southern Europe [33]. This latitudinal gradient may also be related to the increased pattern variability at higher latitudes associated with the jet stream position and temperature gradient. Our findings are consistent with previous research that has related blocking highs and heatwaves to surface fire weather in specific countries and regions of Europe [27,65,66], as well as research finding that synoptic–surface associations were strongest at high latitudes [5,6].

### (c) Persistent positive anomalies and wildfire

We have quantified the pan-European importance of PPAs for wildfire occurrence and burned area, building on previous country-level analyses that have linked anticyclonic blocking to significant wildfire seasons [25,66]. The likelihood of wildfires occurring during PPAs was highest overall for Southern Europe and lowest for Northern Europe. This is tied to differences in the prevalence of wildfire activity versus PPA activity across latitudes. Northern Europe experiences more PPAs than Southern Europe, but has substantially less wildfire activity (high counts of PPA-nofire), while Southern Europe is the opposite (low counts of PPA-nofire), which therefore influences the size of the *OR* denominator and resulting *OR*.

The percentage of burned area associated with PPAs was inversely highest for Northern Europe and lowest for Southern Europe. Burned area in Northern Europe relies on PPA occurrence throughout the fire season; however, in Southern Europe, the highest percentage of PPA-related burned area occurs in October. The majority of burned area during summer occurs under normal seasonal variations in fire weather in Southern Europe as extreme fire weather is not necessarily a requisite for extreme fire behaviour, and this highlights the additional importance of other factors driving large wildfires in Southern Europe such as drought, wind and atmospheric instability [22,51].

While we have demonstrated the significance of PPAs for European wildfires, these PPAs appear to be less important than those observed for Western North American wildfires. Sharma *et al*. [11] found that wildfires larger than 500 ha were on average seven times more likely to occur during PPA conditions. These differences may be related to the lower landscape-level fuel connectivity in Europe compared with the dominance of boreal forest fuels in North America. In Europe, this corresponds with more wildland–urban interfaces and a higher population density with resources for fast fire suppression, which align to limit the extent and number of large fires.

Burned area was largest up to one week following the presence of a PPA. This is likely owing to a lag between the presence of the PPA, the time taken for the high-pressure system to break down when smaller positive geopotential height anomalies may still occur, and the associated drying of fuels that allows subsequent ignitions to take place. The extended peak in the moisture components (FFMC, DC and DMC) of the CFWIS following peak PPA strength observed reflects this. Furthermore, surface wind speeds are likely to be greater at the edge of PPAs, facilitating the spread of wildfires through drier fuels and higher wind speeds, and that may foster rekindling of fires that have not been fully mopped up [25]. The lead–lag graphs show a reduction in wind speeds during peak PPA strength, followed by a delayed return to normal wind speeds. However, surface winds are not well resolved within reanalyses and include topographically forced winds, mixing of mid and surface wind levels and fire-generated downdrafts, making it difficult to elucidate the role of wind speed at the edge of PPAs and during event breakdown. Future research might consider narrowing in on the significance of edge effects on wind and wildfire burned area by looking at the vertical profile of wind through the atmosphere.

We have demonstrated the pan-European importance of PPAs for surface fire weather and wildfire activity, recognizing that our analyses are constrained by the number of fires detectable by MODIS. National datasets that include smaller wildfires may provide a more comprehensive understanding of synoptic–fire relationships for individual countries, but our aim here was to examine PPAs as drivers of wildfire across large regions of Europe and involving multiple countries concurrently. Furthermore, PPAs are part of a larger picture of atmospheric controls on wildfire activity that need to be understood, including the full range of synoptic drivers of wildfire and how they are influenced by larger teleconnections.

### (d) Implications

Our pan-European synthesis of PPA–fire associations demonstrates how PPAs can impact large regions across political borders simultaneously and for extended durations. Our findings have implications for the following areas of research and operations.

#### (i) Extended forecasting ability for wildfire prevention and suppression resource management

The strong association between PPAs and extreme surface fire weather may help to extend forecasting using synoptic feature-based methods in wildfire occurrence prediction models [5,18,22]. The inclusion of synoptic weather patterns to develop early warning systems for dangerous fire weather conditions would enhance wildfire awareness and preparedness, with benefits for prevention planning, public communication of wildfire danger and informing policy, such as implementation of fire bans. Medium-range forecasting of potential PPA-driven wildfire periods may also provide early warning for suppression resource mobilization, for example through the RescEU reserve pool of firefighting assets and coordinating firefighting response to large, long-duration events or high numbers of synchronous events [1]. Across Europe, subsidence under PPAs associated with wildfires leads to poor surface air quality conditions. Smoke air pollution and health impacts from these events can reach densely populated regions of Europe, such as occurred in the 2021 PPA-related wildfires over Greece [67]. Advanced forecasting and awareness of PPA-driven air pollution events may aid early implementation of mitigation strategies, particularly for the densely populated regions of Europe.

#### (ii) Future persistent positive anomalies–fire risk

While we have established the role of PPAs in wildfire activity across Europe, there is uncertainty in how PPAs are expected to change in the future, which contributes to uncertainty in future fire risk [32]. Some research has demonstrated historical increases in blocking frequency and heatwaves over Europe [6,68,69]. Regarding historical trends in PPAs, Sharma *et al*. [11] found a statistically significant expansion of PPAs (but no increase in number or magnitude) over western North America since 1979, driven primarily by warming in the lower atmosphere (thermodynamic changes) rather than dynamic changes.

Projections of future fire weather have mainly examined changes in the FWI with anthropogenic warming, but this does not explicitly account for atmospheric circulation changes and feedbacks [70,71]. Climate change projections have suggested an increase in the frequency, duration and intensity of extreme weather events like heatwaves and drought for Southern Europe [72]. However, climate models typically underestimate the frequency of blocking events [31], and most predict future decreases in blocking frequency. Arctic amplification (AA) thus far has favoured increased double jet stream formation, weakening westerlies and formation of high-pressure blocking cells in the mid-latitudes [6,29,73]. Previous research has linked summertime weakening and northward movement of the polar jet stream to wildfire activity in North America [74] and mid-latitude weather extremes [75]. How these patterns will change with future anthropogenic warning is uncertain, linked to the poor representation of dynamic atmospheric circulation in climate models [73]. Research predicting future fire weather and wildfire regimes should consider dynamic atmospheric circulation changes in addition to thermodynamic changes [76].

#### (iii) Compounding persistent positive anomalies–fire vulnerabilities for high latitude Europe

We observed a latitudinal increase in the percentage of area burned during PPAs. Extended periods of extreme surface fire weather associated with PPAs can lead to drought conditions through land–atmosphere feedbacks, and subsequent wildfires and carbon emissions can have critical ecological and climate consequences [77,78]. Future PPA-driven wildfire burned area in the high latitudes will also be impacted by changing ignition patterns owing to projected increases in summer lightning flashes. Chen *et al*. [77] predicted that, for 1°C of warming, summer lightning flash rates will increase by 40 ± 19% over Arctic tundra and by 23 ± 6% over boreal forest in the circumpolar high-northern-latitudes. Climate change feedbacks may further amplify impacts from PPA-wildfires at high latitudes, through reduced snow cover and declines in permafrost, earlier snow melt and exposure of carbon stores to smouldering combustion under wildfires [78].

## 5. Conclusions

It is critical to understand the mechanisms driving PPA–fire relationships now, to understand how wildfire risk may change in the future. This research presents the first application of PPAs to wildfires in Europe. This event-based paradigm allowed us to track events in space and time and to develop robust statistics to identify when seasonality and location of PPA events can elevate wildfire danger. Our findings highlight opportunities for a more proactive approach to wildfire response at a pan-European scale, through extended forecasting within wildfire occurrence models for effective decision-making during large, extended periods of elevated wildfire danger. Consideration of multi-scale controls on wildfire occurrence and behaviour through both synoptic and surface fire weather may provide a more holistic approach to anticipating and preparing for dangerous wildfire conditions.

## Data Availability

We used the R algorithm of [11] to identify European PPAs, and we direct the reader to this reference for further information. All datasets used in this article are publicly available through the references cited and can be accessed through the web portals: [79] (ERA5 reanalysis data for surface and upper air variables, accessed January 2022) and [80] (EFFIS burned area shapefiles, requested March 2022). Supplementary material is available online [81].
